# Importance of standardizing timing of hematocrit measurement when using cardiovascular magnetic resonance to calculate myocardial extracellular volume (ECV) based on pre- and post-contrast T1 mapping

**DOI:** 10.1186/s12968-018-0464-9

**Published:** 2018-06-28

**Authors:** Henrik Engblom, Mikael Kanski, Sascha Kopic, David Nordlund, Christos G. Xanthis, Robert Jablonowski, Einar Heiberg, Anthony H. Aletras, Marcus Carlsson, Håkan Arheden

**Affiliations:** 1 Department of Clinical Physiology, Clinical Sciences, Lund University and Lund University Hospital, Getingevägen 3, 221 85 Lund, Sweden; 2 Laboratory of Computing, Medical Informatics and Biomedical – Imaging Technologies, School of Medicine, Aristotle University of Thessaloniki, Thessaloniki, Greece

**Keywords:** Extracellular volume, ECV, T1 mapping, Hematocrit

## Abstract

**Background:**

Cardiovascular magnetic resonance (CMR) can be used to calculate myocardial extracellular volume fraction (ECV) by relating the longitudinal relaxation rate in blood and myocardium before and after contrast-injection to hematocrit (Hct) in blood. Hematocrit is known to vary with body posture, which could affect the calculations of ECV.

The aim of this study was to test the hypothesis that there is a significant increase in calculated ECV values if the Hct is sampled after the CMR examination in supine position compared to when the patient arrives at the MR department.

**Methods:**

Forty-three consecutive patients including various pathologies as well as normal findings were included in the study. Venous blood samples were drawn upon arrival to the MR department and directly after the examination with the patient remaining in supine position. A Modified Look-Locker Inversion recovery (MOLLI) protocol was used to acquire mid-ventricular short-axis images before and after contrast injection from which motion-corrected T1 maps were derived and ECV was calculated.

**Results:**

Hematocrit decreased from 44.0 ± 3.7% before to 40.6 ± 4.0% after the CMR examination (*p* < 0.001). This resulted in a change in calculated ECV from 24.7 ± 3.8% before to 26.2 ± 4.2% after the CMR examination (*p* < 0.001). All patients decreased in Hct after the CMR examination compared to before except for two patients whose Hct remained the same.

**Conclusion:**

Variability in CMR-derived myocardial ECV can be reduced by standardizing the timing of Hct measurement relative to the CMR examination. Thus, a standardized acquisition of blood sample for Hct after the CMR examination, when the patient is still in supine position, would increase the precision of ECV measurements.

## Background

Cardiovascular magnetic resonance (CMR) has evolved as the imaging reference standard for diagnosis of a variety of myocardial pathologies due to the versatility with which myocardial tissue can be characterized. For diffuse pathology such as diffuse fibrosis, inflammation, edema or myocardial storage disease, parametric mapping techniques such as T1-, T2- and T2* mapping of the myocardium have shown great potential. T1-mapping can also be used to assess myocardial extracellular volume fraction (ECV). The use of T1 mapping for calculating myocardial ECV in vivo in an experimental setting was first shown by Arheden et al. [1, 2] by generating T1 maps of myocardium and blood before and after contrast injection and applying the following equation:

1 $$ Myocardial\kern0.5em ECV\kern0.5em =\kern0.5em \left(1- Hct\right)\frac{1/ Myocardial\kern0.5em T{1}_{post\kern0.5em contrast}-1/ Myocardial\kern0.5em T{1}_{pre\kern0.5em contrast}}{1/ Blood\kern0.5em T{1}_{post\kern0.5em contrast}-\kern0.5em 1/ Blood\kern0.5em T{1}_{pre\kern0.5em contrast}} $$

Thus, the calculated ECV is directly proportional to the 1-hematocrit (Hct) of the blood, which is defined as the volumetric percentage of red blood cells in whole blood. A change in Hct would therefore change ECV as derived from Eq. [1]. The first clinical implementation of CMR-derived myocardial ECV was recently described by Ugander et al. [3].

**Fig. 1:**
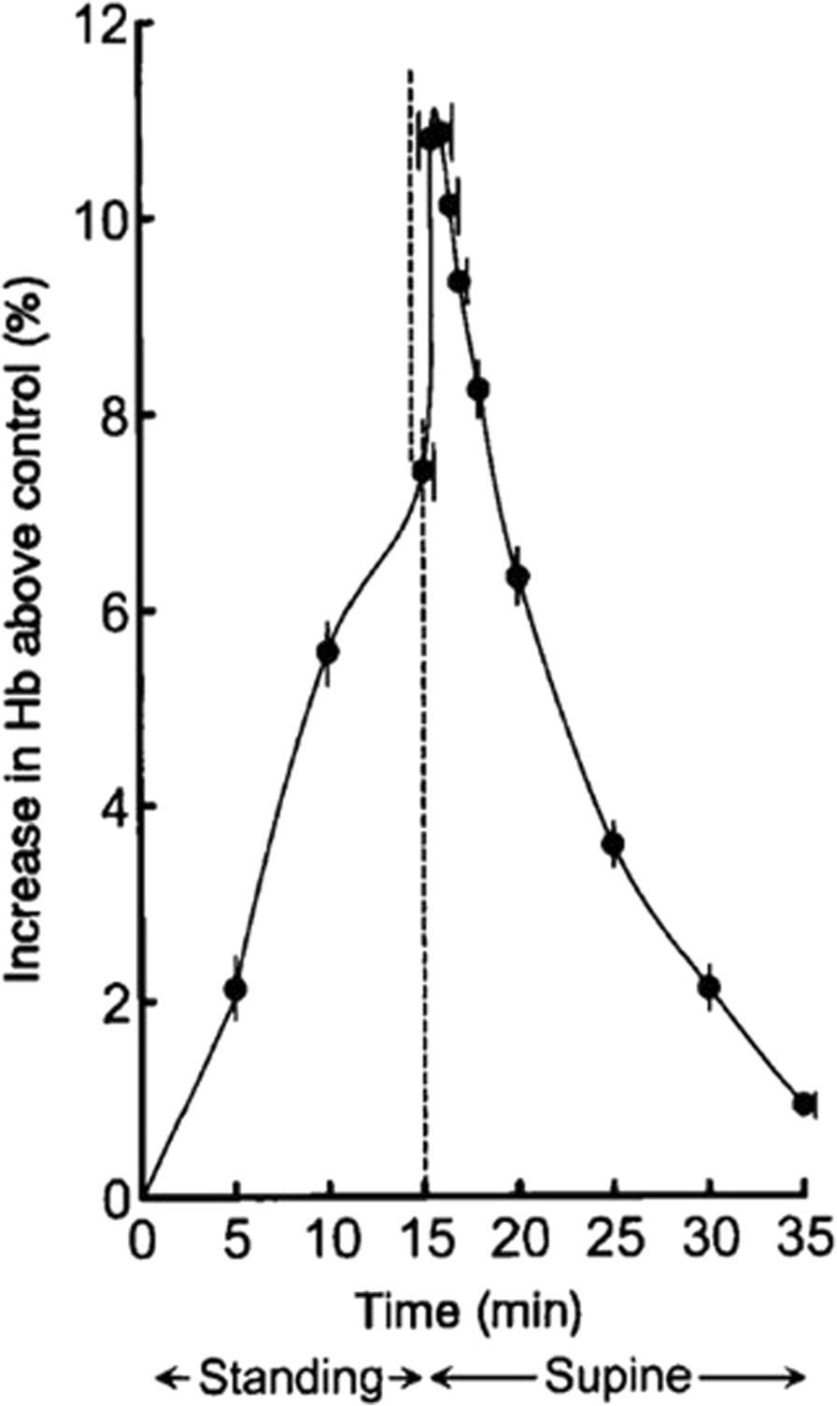
From Fig. 1 in Lundvall et al. [5]. Collected data (mean ± SE; 16 experiments) on arterial haemoconcentration (Hb; percent increase above control) during and after 15 min of quiet standing. Note that Hb showed only moderate increase at the end of the period of standing but a rapid and marked further increase when the supine body position was resumed, signifying that blood collected in the erect posture markedly underestimated the ‘true, overall’ haemoconcentration. (Reproduced with permission)

It has previously been shown that there is a significant postural-dependent change in Hct levels [4, 5]. In the clinical context this phenomenon is known as postural pseudoanemia, as interstitial fluid from the lower extremities re-enters the blood pool when transitioning from standing to supine position, thereby lowering the Hct and consequently increasing the calculated ECV according to Eq. [1]. For example *Lundvall* et al. [5] demonstrated that changes in posture result in fluctuations of hemoglobin concentrations up to 11% (Fig. 1). Thus, Hct may change significantly depending on body position and therefore vary depending on when the blood sample is taken in relation to an examination performed with the patient in supine position. To what extent the timing of Hct measurement affects the CMR-based calculations of ECV in a clinical context is not known. The prognostic significance of ECV has been shown in patients with heart failure with decreased [6] and preserved [7] ejection fraction to be superior to ejection fraction. Therefore it is probable that ECV will gain importance as an outcome variable in future clinical trials. In the design of such trials, the knowledge of the effect on confounders such as Hct variation is of importance.

Therefore, the aim of this study was to test the hypothesis that there is a significant increase in calculated ECV value if the Hct is taken after the CMR examination in supine position compared to before the examination when the patient arrives at the MR department.

## Methods

The study was approved by the regional ethics committee and all subjects provided written informed consent. A total of 43 consecutive patients including various pathologies and normal findings (Table 1) were included during approximately 3 weeks in June 2017, thereby representing a non-selected clinical patient population. Venous blood samples were drawn from an antecubital vein both at arrival to the MR department and directly after the examination when the patient was still in supine position. The blood sampling prior to the CMR examination was not standardized, but performed according to everyday clinical routine, which means that some patients walked into the MR-department with the pre-examination blood sample taken without spending time in the waiting room, whereas other patients spent a variable amount of time sitting in the waiting room before the pre-examination blood sample was drawn. Hematocrit was measured on-site using an i-STAT blood analyzer (Chem8+ cartridge, Abbott Laboratories, Chicago, Illinois, USA).

**Table 1:** Patient characteristics

Patient characteristics				
Number of patients			43	
Gender (f/m)		9	/	34
Age (years)		55	±	15
Height (cm)		177	±	8
Weight (kg)		86	±	18
BSA (m^2^)		2,0	±	0,2
LV EDV (ml)		202	±	81
LV ESV (ml)		108	±	70
LV SV (ml)		94	±	27
EF (%)		50	±	12
HR (bpm)		70	±	14
Diagnosis*				
Non-ischemic DCM			11	
IHD			7	
Myocarditis			8	
HCM			2	
ARVC			2	
PAH			2	
Sarcoidosis			2	
Non-specific			5	
No pathology			7	

*ARVC* = arrhythmogenic right ventricular cardiomyopathy, *BSA* = body surface area, *DCM* = dilated cardiomyopathy, *EDV* = end-diastolic volume, *EF* = ejection fraction, *ESV* = end-systolic volume, *HCM* = hypertrophic cardiomyopathy, *HR* = heart rate, *IHD* = ischemic heart disease, *LV* = left ventricular, *PAH* = pulmonary arterial hypertension, *SV* = stroke volume *Three patients had dual pathology

### MR imaging and analysis

#### Image acquisition

All patients underwent CMR on a MAGNETOM Aera 1.5 T scanner (Siemens Healthineers, Erlangen, Germany) using a 30-channel coil (body array and spine array). A Modified Look-Locker Inversion recovery (MOLLI) protocol based on a prototype sequence with an acquisition scheme of 5 s(3 s)3 s was used to acquire a midventricular short-axis image before injection of 0.2 mmol/kg Gd-DOTA (Dotarem, Guerbet, Roissy, France). A MOLLI protocol with an acquisition scheme adjusted for post-contrast imaging of 4 s(1 s)3 s(1 s)2 s was then repeated in the same short-axis view approximately 15–20 min after contrast injection. Motion-corrected T1 maps were derived from both the pre- and post-contrast MOLLI images. For all patients, cine balanced steady-state free precession (bSSFP) images as well as late gadolinium enhancement (LGE) were acquired in short-axis (covering the entire left ventricle) and in the standard 2-, 3-, and 4-chamber long-axis views. No patients were given fluids during the MR examination.

#### Image analysis

**Fig. 2:**
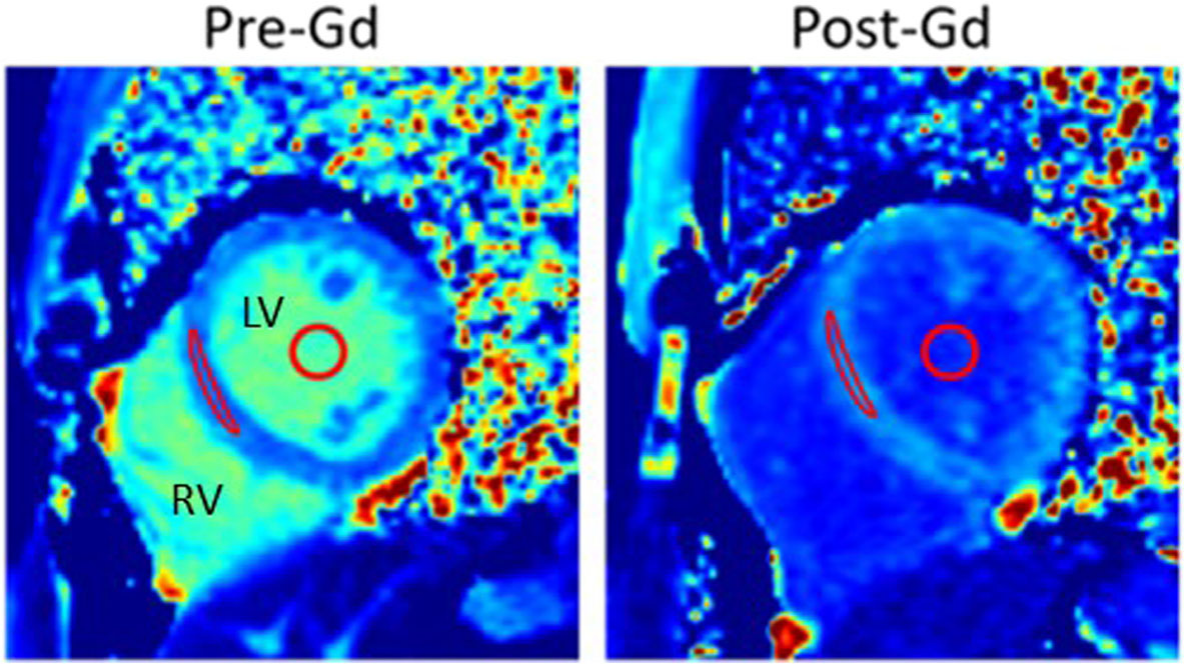
Mid-ventricular left ventricular short-axis T1 maps acquired pre- and post-Gd contrast injection in one subject. Region of interests in which T1-values were measured in the septal wall and in the blood pool are indicated in red. The extracellular volume fraction (ECV) was calculated based on these T1 measurements related to Hct according to Eq. [1]. Gd = gadolinium, Hct = hematocrit, LV = left ventricle, RV = right ventricle

All images were analyzed using the software Segment, version 2.0 R5453 (http://segment.heiberg.se) [8]. T1 measurements were performed by drawing a region of interest in LGE-negative myocardium as well in the blood pool in both pre- and post-contrast T1 maps (Fig. 2). T1-values were used to calculate myocardial ECV according to Eq. [1], both with the Hct sampled before and after the examination. LGE images were used to detect regional myocardial injury to be avoided for the region of interests drawn in the T1 maps. Cine bSSFP short-axis images were used to quantify left ventricular function and planimetric volumes.

### Statistics

Statistical analysis was performed using GraphPad Prism (v7.01, GraphPad Software, La Jolla, California, USA). Values are provided as mean ± SD. To test for differences in Hct and ECV before and after the examination, a two-tailed paired parametric t-test was applied after excluding deviation from normal distribution with a D’Agostino-Pearson test. An unpaired t-test was applied to test for difference in the degree of change in Hct and ECV between sexes. An ANOVA test was applied to test for difference in the degree of change in Hct and ECV between pathology subgroups and a Pearson’s correlation coefficient was used to assess the correlation between age, body weight, BSA and the degree of change in Hct and ECV. A *p* value of < 0.05 was considered to indicate statistical significance.

## Results

Patient characteristics including pathologies are shown in Table 1. Data were excluded for one patient, who presented with cold autoimmune hemolytic anemia, therefore a reliable Hct reading could not be ensured.

**Fig. 3:**
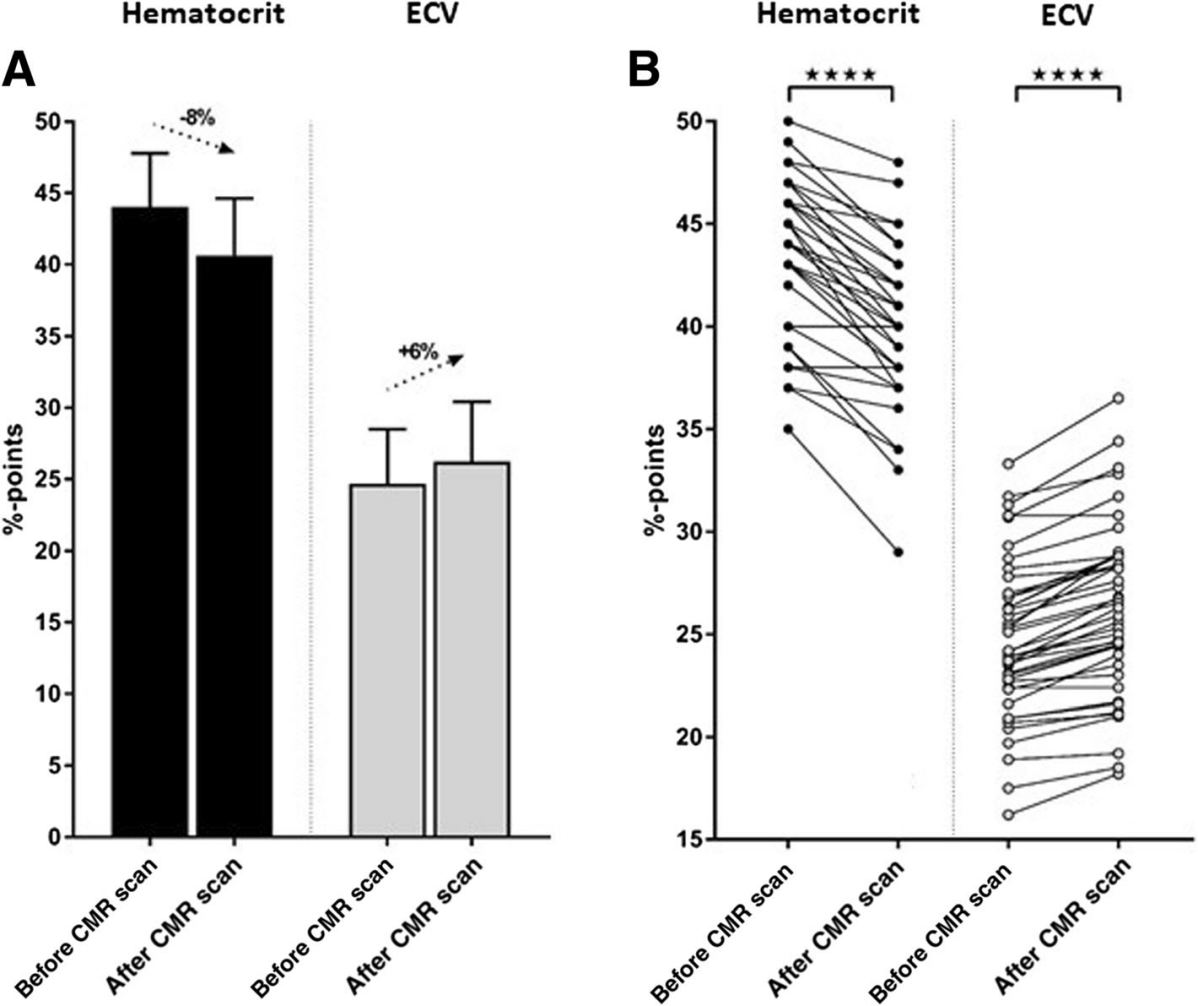
The change in Hct and ECV between before and after a CMR examination. **a**) The mean Hct (black bars) and mean ECV (grey bars) before and after the CMR examination. Error bars indicate standard deviation. **b**) Individual change in Hct (filled circles) and ECV (open circles) for all 43 subjects. Note that all patients decreased in Hct after the examination compared to before except for two patients who remained the same. **** indicates *p* < 0.001. CMR = cardiovascular magnetic resonance, ECV = extracellular volume fraction, Hct = hematocrit

The average Hct before CMR examination was 44.0 ± 3.7%. After the CMR examination hematocrit decreased to 40.6 ± 4.0% (*p* < 0.001; Fig. 3a). The average scan time for the full CMR protocol, which corresponds to the minimal interval between drawing of the pre- and post-examination blood samples, was 52 ± 16 min (range 32–113 min). The sampled Hct values translated into an average calculated ECV of 24.7 ± 3.8% (before) and 26.2 ± 4.2% (after), respectively (*p* < 0.001; Fig. 3). No differences in the degree of change in Hct or resulting ECV was seen between sexes or individual pathologies (data not shown). Furthermore, no correlations between age, body weight or BSA or type of pathology and the degree of change in hematocrit or resulting ECV between both sampling time-points could be identified (data not shown). Note that all patients decreased in Hct after the CMR examination compared to before except for two patients where Hct remained the same (Fig. 3b).

The extra time the patient spent on the scanner table due to blood sampling after the CMR examination was approximately 2 min.

## Discussion

This study shows the importance of standardizing the timing at which Hct is sampled in relation to the CMR examination during which T1 maps for ECV calculation are acquired. To decrease variability in ECV related to variation in Hct measurements, blood should preferably be sampled after the CMR examination when the patient is still in supine position. If the blood sample must be taken before the CMR examination, the patient should be in supine position at least for 25 min before the blood is drawn based on results from Lundvall et al. [5].

### Change in Hct due to change in posture

Hematocrit changed by 8% when comparing blood samples drawn before (arriving at the MR department) and after CMR examination (still in supine position). This change in Hct due to change in body posture is somewhat smaller compared to what Jacob et al. [4] showed in healthy subjects where a change of 11.0% was observed comparing supine position to 30 min of standing. The reason for the smaller change observed in the present study could partly be explained by the fact that the study by design was executed in a non-controlled clinical setting to reflect the Hct changes observed in this context. Thus, the present study included both patients entering the MR-department by foot and having the pre-examination blood sample taken without spending time in the waiting room, whereas other patients spent a variable amount of time sitting in the waiting room before the pre-examination blood sample was drawn. Therefore, the pre-examination conditions were less controlled than for the subjects in the study by Jacob et al. [4]. Lundvall et al. showed an even greater change in Hct (12.4%) between supine position and standing when posture change was studied under even more controlled circumstances using a tilt table in healthy subjects to minimize the influence of the muscle pump in the lower limbs on the amount of extracellular fluid [5]. Furthermore, Lundvall et al. [5] showed that plasma volume and thereby the Hct returns to baseline levels after approximately 20–25 min in supine position after the subjects had been tilted to an upright position (85°) for 15 min. Thus, patients who have undergone a CMR examination (usually > 25 min) in supine position are most likely in steady state with regard to plasma volume related to body posture changes. Current recommendation from Society for Cardiovascular Magnetic Resonance and European Association of Cardiovascular Imaging (SCMR/EACVI) [9] state that for ECV calculations with T1 mapping, Hct should be taken immediately before the CMR scan or, if that is not possible, within 24 h of the CMR scan. Based on the findings in the present study, these recommendations might need to be changed in the next revision of these guidelines, recommending blood sampling in supine position at the end of the examination. The short extra time (approximately 2 min) that the patient needs to spend on the scanner table to have Hct taken in supine position after the CMR examination is not believed to significantly affect the throughput of patients since the patient already has an intravenous access for contrast agent administration.

### Factors affecting variability in calculation of ECV

The present study shows that CMR-derived ECV changes by 6.3% (range 0–14.6%) when Hct is taken before instead of after the CMR examination. Previous studies have shown little variation in CMR-derived ECV when considering intra-study variability. It has been shown that ECV values are stable over a wide range of time after contrast injection for the post-contrast T1 measurements [10, 11]. Furthermore, *Kawel* et al. have shown that CMR-derived ECV is similar between 1.5 T and 3 T, with small but systematic differences between different locations in the left ventricular myocardium and depending on when during the cardiac cycle images are acquired [12].

### The use of CMR-derived ECV as a biomarker for therapeutic efficacy

There are currently several ongoing clinical trials using CMR-derived myocardial ECV for assessment of therapeutic efficacy or for describing the evolution of myocardial disease over time. The change in CMR-derived ECV (6.3%) related to Hct being taken before or after the CMR examination found in the present study is similar to the differences found between normal controls and patients with heart failure (7.4%) [13] or tetralogy of Fallot (8.0%) [14]. Thus, it is important to ensure standardized sampling of blood for Hct assessment used to derive ECV to be used as an outcome measure in clinical trials. If a study design results in a systematic difference in body posture in the different treatment arms or disease states when blood is sampled there is a risk for both type I and type II errors. Examples of possible situations that could introduce a bias would be if patients are transported in bed to the CMR examination in the treatment group whereas the controls are not, resulting in differences in plasma volume related to different body posture. Alternatively, the treatment group may already have taken their blood samples for other purposes before the CMR examination whereas patients in the control group may not. This bias can be avoided if Hct is always taken after the CMR examination when the patient or study subject remains in the supine position.

Another way to avoid this bias would be if ECV calculations could be performed without the need for blood sampling. It has recently been proposed that T1 mapping can be used to determine a synthetic Hct derived from the longitudinal relaxation rate of blood [15, 16]. Even though they showed a significant correlation (r^2^ = 0.51) between longitudinal relaxation rate of the blood and Hct measured from venous blood, there may be significant difference in actual ECV and synthetic ECV for individual patients, specifically pediatric patients and young adults [17], affecting the ability to detect relatively small differences in ECV between normal and diffuse disease or treated vs non-treated subjects.

## Conclusions

This study shows that the variability in myocardial ECV calculations by CMR can be reduced by standardizing the timing of Hct measurement in relation to the CMR examination. Thus, a standardized acquisition of blood sample for Hct after the CMR examination, when the patient is still in supine position, would increase the precision of ECV measurements.

## Abbreviations

bSSFP: balanced steady state free precession
CMR: Cardiovascular magnetic resonance
ECV: Extracellular volume fraction
Hb: Hemoconcentration
Hct: Hematocrit
LGE: Late gadolinium enhancement
MOLLI: Modified Look-Locker inversion recovery
